# Mechanical Characteristics of Glass-Fiber-Reinforced Polyester Composite Materials

**DOI:** 10.3390/ma18153595

**Published:** 2025-07-31

**Authors:** Ioan Milosan, Tibor Bedo, Camelia Gabor, Mihai Alin Pop

**Affiliations:** Department of Materials Science, Transilvania University of Brasov, 29 Eroilor Blvd., 500036 Brasov, Romania; milosan@unitbv.ro (I.M.); camelia.gabor@unitbv.ro (C.G.); mihai.pop@unitbv.ro (M.A.P.)

**Keywords:** composites, fiber glass, mechanical properties, internal architecture

## Abstract

Fiber-reinforced composites are gaining more importance across different fields such as aeronautics, automotives, high-performance sporting equipment, etc., where decreasing weight while improving mechanical properties of polymers is fundamental. This article explores the mechanical behavior of fiber-reinforced polyester composite materials, highlighting their advantages and applications in various industrial fields. Usually, composite materials consist of a polyester matrix reinforced with different types of fibers, such as glass, carbon, or Kevlar, which provide superior mechanical characteristics. This study analyzed the tensile strength, bending resistance, and resilience of glass fiber composites, emphasizing the importance of proper fiber selection and manufacturing processes. These materials stand out for their excellent strength-to-weight ratio and are widely used in the fabrication of tanks in various industries. Experimental results demonstrated tensile strength (Rm) around 115 MPa, Shore D hardness values of 88 units, and impact toughness (resilience) of 2.7 J/cm^2^. Based on the composite materials’ behavior in testing, the article further offers practical recommendations for the effective deployment of these composites in the fabrication of various types of industrial reservoirs.

## 1. Introduction

Glass-fiber-reinforced composites (GFRC) represent a class of heterogeneous composite materials in which glass fibers, known for their excellent mechanical properties, are embedded within a polymer matrix, whether via thermosetting or thermoplastic. This configuration creates a multiphase system that enhances stiffness, mechanical strength, and durability, all while maintaining relatively low production costs. The mechanical performance of GFRC materials is influenced by a range of microstructural and interfacial factors, including the fiber volume fraction, elastic modulus, fiber strength, and distribution and orientation of the reinforcing fibers. Together with the quality of the fiber–matrix interface, these elements significantly impact load transfer efficiency and degradation behavior.

Studying these properties implies analyzing the influence of various parameters, such as fiber type, fiber orientation, fiber volume content, matrix type, and processing conditions. A thorough understanding of how these factors affect the mechanical behavior of glass fiber composites enables the optimization of structural design for enhanced performance and durability under diverse operating conditions.

The outstanding characteristics of glass-fiber-reinforced composites have made them essential materials in numerous industries, including automotive, chemical, petrochemical, construction, aerospace, and biomedical engineering [1,2,3]. This widespread adoption has prompted extensive research efforts, within both specialized research institutes and academic institutions, aimed at investigating the key factors influencing the performance of these materials. Numerous researchers have examined the effects of reinforcement elements, both in terms of their composition and geometry, on the properties of composite materials [1,4,5]. Some studies have focused specifically on fiber-reinforced composites [2,6,7,8,9], with glass fibers being one of the most commonly utilized reinforcement elements in this context [6,7,9].

Complex microstructural phenomena (such as delamination, microcrack propagation within the matrix, and interfacial debonding) directly affect the mechanical response of a composite under both static and dynamic loading, including tensile, flexural, torsional, and impact resistance.

The superior mechanical properties of these composites position them as viable alternatives to metallic materials across a range of applications and justify the continuous advanced research in this area. Numerous studies have addressed the general mechanical properties of these composites [3,10,11,12,13,14], while others have focused more narrowly on specific characteristics such as tensile strength [15], resilience [16], and tribological behavior [17].

In addition to the reinforcement phase, the polymer matrix plays a critical role in enhancing the mechanical performance of fiber-reinforced composites [18,19,20,21]. Furthermore, fiber–matrix interactions are essential in determining fatigue behavior and material durability under varying environmental conditions, including humidity, temperature fluctuations, and chemical exposure. Experimental investigations employing standardized testing methodologies are complemented by numerical analyses (finite element method) and advanced microstructural characterization techniques (scanning electron microscopy, computed tomography) aimed at developing a detailed understanding of mechanical behavior and optimizing processing parameters [22].

This study focuses on the investigation of the fundamental mechanical properties of glass-fiber-reinforced composites, examining the influence of processing parameters and highlighting potential industrial applications of these versatile materials. Thus, it was demonstrated that composites with different internal architectures that were not previously reported in the literature exhibited superior mechanical behavior.

The present study is grounded, among other factors, in the rapidly expanding trends concerning the use of these types of materials across a wide range of advanced industrial sectors [23], as well as in the inherent limitations associated with conventional metallic materials [24], bringing novelty through the internal structure (architecture) of the composite materials developed and investigated.

## 2. Materials and Methods

The composite materials studied herein were supplied by SC UPRUC POL SA Făgăraș, Făgăraș, Romania. These consisted of glass-fiber-reinforced polyesters intended for the fabrication of tanks utilized in the chemical, petrochemical, food processing, agricultural, and other industrial sectors.

Four types of composite materials with different architectures were investigated. To ensure the reproducibility of the results and experimental reliability, a sufficient number of test specimens were prepared for each composite variant. Specimens were prepared for tensile, flexural, hardness, and resilience testing. 

**Figure 1:**
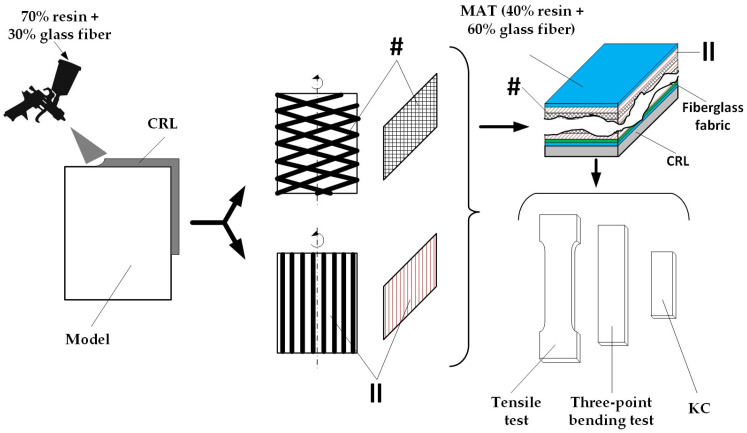
Composite material manufacturing flow. CRL—chemical-resistant lining, #—crosswise configuration layers, ||—parallel configuration layers.

**Figure 2:**
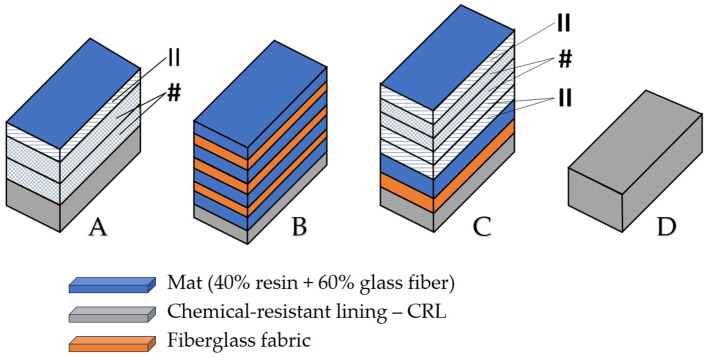
Architectural configuration of the tested composite materials. #—crosswise-configuration layers, **||**—parallel-configuration layers.

The test specimens were cut and prepared from composite plates manufactured by S.C. UPRUC POL S.A. Făgăraș, Romania. Their fabrication followed a series of well-defined steps, as outlined in the technical production specification sheet shown in Figure 1. The tested materials, along with their architectural configuration, are presented in Table 1 and Figure 2.

First, a layer of base material with a thickness between 2.2 and 2.4 mm was sputtered onto a mold using a spray gun. The base material consisted of 70% resin and 30% glass fiber. The material in this state is denoted as CRL (chemical-resistant lining).

Depending on the intended application of the final product, the manufacturing process continued as follows:

- For tank lids, the plate was used in its initial form without additional layers, corresponding to material D;
- For tanks designated for the food industry, the initial product, from the first step, was placed on a filament winding machine to apply additional layers in either a crosswise (#, 1.6 mm thickness) or parallel (||, 1.0 mm thickness) configuration. The resin used in this case was isophthalic resin (specifically formulated for food industry applications), selected to avoid chemical contamination of the stored liquids. This was specific to material A;
- For tanks used in the chemical, petrochemical, and related industries, the resin applied in additional crosswise (#) or parallel (||) layers was orthophthalic resin. The added material had a composition of 40% resin and 60% glass fibers. The material layer had a thickness of 0.9 mm (M), while a fabric layer had a thickness of 1.2 mm (T). This method was used for materials B and C.

The entire manufacturing process took place at ambient workshop temperature, while the curing time was 10 h.

Mat or Stratimat refers to a sheet of glass fibers used as a base layer, combined with woven fabric that provides directional strength and distributes mechanical loads. These are bonded together by a resin matrix, which both reinforces and unifies the layers, imparting rigidity and cohesion to the entire laminate structure. Because of limitations in the fabrication process, producing control specimens composed solely of resin is not practical, as they tend to develop cracks during curing. This constraint highlights one of the key reasons for incorporating reinforcing fibers, namely, to enhance structural integrity and enable the formation of specimens with adequate thickness and mechanical stability.

Initial specimen preparation was performed using a PROXXON LU-6868 Wecker cutting machine (200 W power, PROXXON GmbH, Föhren, Germany).

Tensile and flexural tests were conducted using a WDW-150S universal testing machine (Jinan Testing Equipment IE Corporation, Jinan, China) with a load capacity between 0–150 kN in order to evaluate the mechanical properties. Testing procedures followed the standards of ISO 527 [25] and ISO 14130 [26]. Hardness measurements were performed using a Sauter HBD 100-0 hardness tester (SAUTER GmbH, Villingen-Schwenningen, Germany), while resilience was determined using a Charpy impact tester with a potential energy of 150 J. For each type of mechanical test, five specimens were prepared from each material variant. A Leica EMSPIRA 3 3D optical microscope (Leica Microsystems Ltd., Heerbrugg, Switzerland) was used to capture both the fracture patterns after impact testing and the structural architectures of the specimens. Further structural image analysis was conducted with the ImageJ 1.54g software package.

## 3. Results and Discussion

### 3.1. Shore Hardness

**Figure 3:**
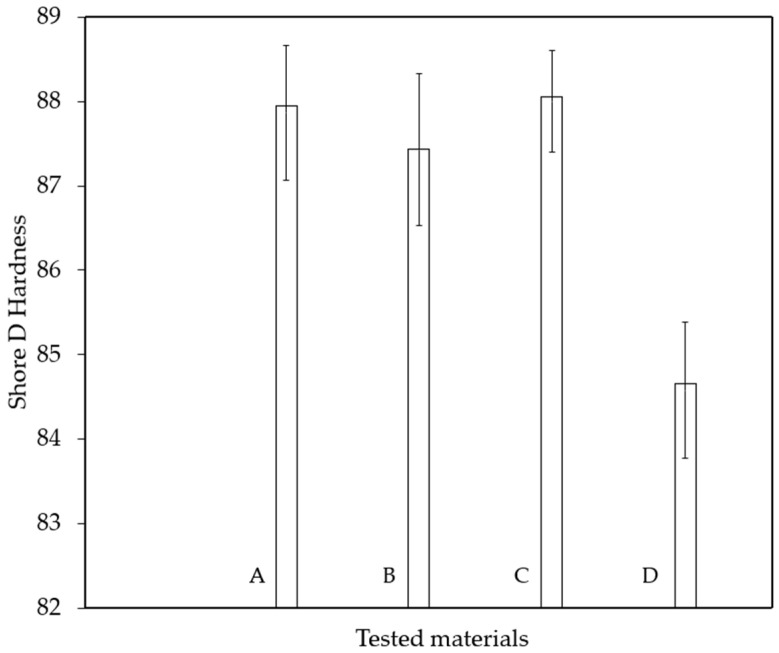
Shore D hardness mean-value for each group of material.

Before preparing the specimens for mechanical testing, the hardness of the base material was measured. Measurements were taken at nine evenly distributed points across the material’s surface. The results of these determinations are presented in Figure 3.

The Shore D hardness test results for the four glass-fiber-reinforced composite materials were relatively close. However, slight variations were present that can be interpreted in terms of the mechanical and structural characteristics of the materials.

As shown in Figure 3, material C exhibited the highest hardness value (88 units), followed closely by materials A and B. The differences among these three materials (C, A, B) were minimal and can be considered practically insignificant.

Material D, on the other hand, showed a noticeably lower hardness (84.58 units), approximately 3.5 units less than the hardest material (C). This notable difference may be attributed to a reduced glass fiber content, the presence of internal defects, or a lower degree of material consolidation.

Materials A, B, and C displayed similar mechanical behavior in terms of hardness and are suitable for applications requiring high and consistent surface hardness. The lower hardness of material D may negatively impact its performance in structural or wear-related applications.

### 3.2. Tensile Testing

**Figure 4:**
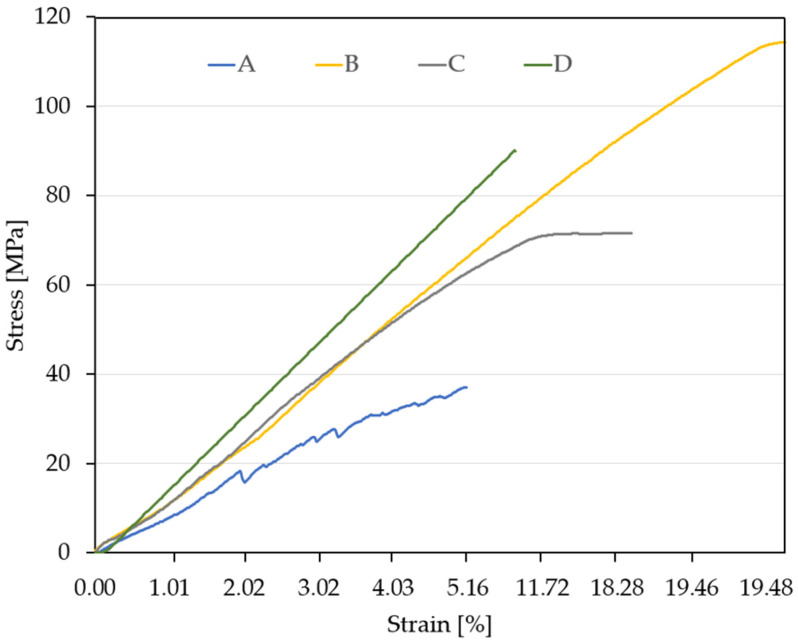
Stress–strain diagram for the four analyzed materials.

Prior performing the tensile tests, the cross-sectional dimensions of the cut specimens—specifically the width and thickness—were precisely measured. These values were used as input parameters for the testing machine software (JinanTE Max Test, version 5.04). Following the tensile tests, representative stress–strain curves were generated for each material. The comparative results are presented in Figure 4.

An analysis of the stress–strain curves following a typical parametric approach [27], for the four types of materials yielded several observations. Material A (blue curve) exhibited highly ductile behavior, with a gradual (slow rate) increase in stress relative to strain. The irregular shape of the curve may suggest internal structural inhomogeneity or nonuniform plastic deformation behavior. It reached a maximum tensile strength of approximately 40 MPa—the lowest value among the four materials—and displayed a low elastic modulus, as indicated by the shallow slope of the curve. The material underwent significant deformation before failure. It is suitable for applications where flexibility and energy absorption are desired rather than high strength.

Material B (yellow curve) demonstrated almost perfectly linear elastic behavior up to the point of failure, achieving a maximum tensile strength of 114 MPa—the highest recorded value. It possessed a very high elastic modulus, as evidenced by the steep slope of the curve. The material also showed considerable total deformation, suggesting a rare combination of stiffness and ductility. It is ideal for load-bearing structures or high-performance components requiring both strength and rigidity.

Material C (gray curve) showed a distinct mechanical response: an initially linear region followed by a plateau, indicating yielding or the onset of plastic deformation. Its maximum tensile strength was approximately 70 MPa, with a moderate elastic modulus. This material is appropriate for applications involving moderate loads where a balance between strength and deformability is important.

Material D (green curve) had very high stiffness and a maximum tensile strength of approximately 90 MPa, with the steepest slope among all the curves, reflecting the highest elastic modulus. A steep initial increase at low strain followed by a sudden drop in the curve suggests a brittle failure mechanism. The low deformation at failure indicates a brittle material, suitable for components where minimal deformation is critical. However, this brittleness may pose challenges in applications involving shocks or variable loading conditions.

Table 2 provides a side-by-side comparison of the four materials derived from the tensile testing data.

### 3.3. Three-Point Bending Test

**Figure 5:**
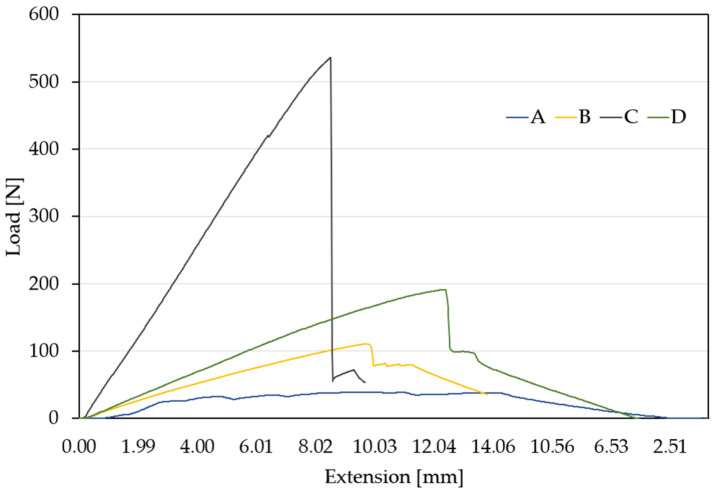
Load–extension curves for the four analyzed materials.

As with the tensile tests, representative curves were established for each material type following the flexural tests, and the comparative results are presented in Figure 5 and Table 3.

An analysis of the flexural behavior of the four composite materials revealed several key conclusions. Material A (blue curve) exhibited a low load-bearing capacity—the curve flattened and remains relatively constant after a small initial peak. It showed a large deflection and a ductile failure mode, continuing to extend even after the applied load decreased. This flexural behavior makes it suitable for components requiring flexibility but not designed to sustain high loads.

Material B (yellow curve) withstood a maximum load of approximately 130 N and showed moderate-to-high deflection. Its failure mode was semiductile, with a gradual onset of failure. This material is appropriate for structures subjected to moderate bending loads.

Material C (gray curve) displayed very low deflection and a brittle failure mode—the curve terminated abruptly without significant deformation. It is suitable only for applications where deformation is not permissible and brittle behavior is acceptable.

Material D (green curve) showed large deflection—indicating its ability to absorb energy even after the onset of failure—and a pseudobrittle failure mode. While the material did fail, the process was not sudden, allowing for some load redistribution. This makes it well-suited for structures subjected to high loads where a degree of deformation is tolerable, such as energy-dissipating or protective elements.

### 3.4. Impact Testing

**Figure 6:**
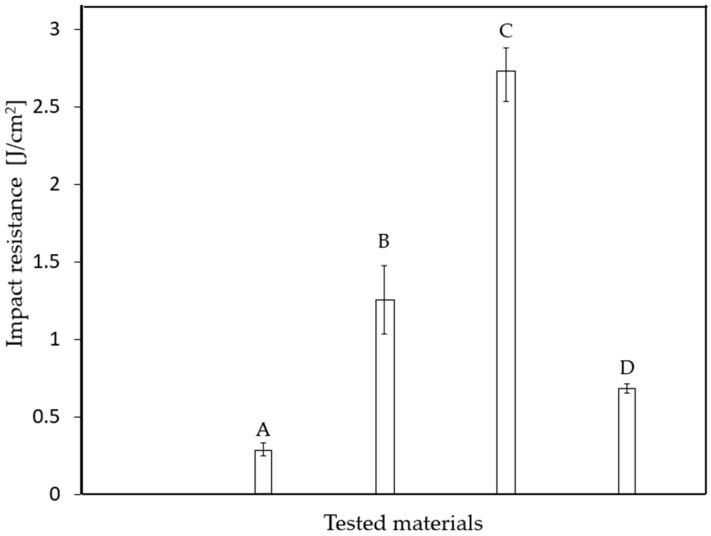
Impact toughness (KC) of the analyzed composite materials.

Five specimens were prepared from each material type and tested under impact loading. The results of the resilience tests for all four composite materials are presented in Figure 6 and Table 4 (each value represents the average of five measurements).

**Figure 7:**
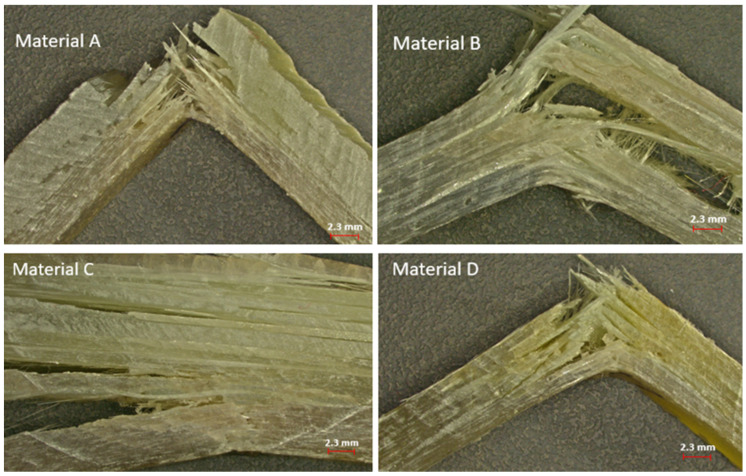
Fracture morphology of the analyzed materials following impact loading.

Fracture patterns observed after impact testing are illustrated in Figure 7, with representative images selected for each material type. A closer analysis of the fracture behavior across the four materials revealed several noteworthy conclusions:

- Material A exhibited a brittle fracture mode characterized by sudden failure and rapid crack propagation. The fibers were transversely broken and appeared to have absorbed minimal energy. This composite displayed low toughness and limited energy absorption likely due to a brittle matrix or poor fiber–matrix adhesion.
- Material B showed a semiductile fracture behavior featuring a combination of broken and drawn fibers, visible delamination, and interlayer separation. Partially elongated fibers indicated greater energy absorption than material A. Composite B demonstrated more balanced properties and improved fiber–matrix adhesion. It was tougher and more damage-tolerant than material A.
- Material C presented an intermediate behavior between brittle and tough. While the matrix contributed to a relatively abrupt fracture, the glass fibers played a role in dissipating impact energy through fiber breakage and pull-out. This material cannot be classified as fully brittle, yet it did not exhibit high toughness either. The fiber layout—unidirectional or layered—indicated mechanical anisotropy, meaning that the mechanical response varies with the direction of loading. The observed fiber pull-out and nonuniform fracture suggest partial energy absorption through microcracking and fiber–matrix decohesion.
- Material D displayed a distinctly ductile failure mode. The glass fibers were stretched over significant distances, with many remaining intact or only slightly damaged. Fracture required considerable energy, and fiber pull-out from the matrix was evident. This material was tough, with strong fiber–matrix adhesion and a high capacity for absorbing impact energy.

Analyzing the images of the four glass-fiber-reinforced composite materials revealed notable differences in fracture behavior and fiber pull-out length. In the case of material A, the fracture was localized in a smaller region, and the glass fibers were distinctly detached, showing a relatively short pull-out length. This type of fracture suggests brittle behavior, where the fibers experienced limited elongation before failure—an indication of reduced resistance to wear and fracture propagation. For material B, the fracture was significantly more diffuse, with a broader fracture zone and more extensive fiber pull-out. The fibers were partially broken and appear to have been pulled out over a greater length compared with material A, indicating a more progressive fracture and a higher elongation capacity prior to failure. This behavior suggests that material B was more ductile than material A. Material C showed a fracture that was even more widespread, with fibers showing substantial pull-out lengths. Compared with the previous materials, material C exhibited the longest fiber extraction, which indicated enhanced ductility and a greater ability to absorb energy prior to fracture. For Material D, similarly to Material A, the fracture was concentrated in a small area, and the fibers were pulled out over a relatively short length. However, some fiber elongation was observed before failure, representing an intermediate fracture performance relative to the other materials.

When considering the overall impact behavior of the four materials, each intended for industrial tank applications, a set of general conclusions can be drawn, as summarized in Table 5.

### 3.5. Internal Structure

**Figure 8:**
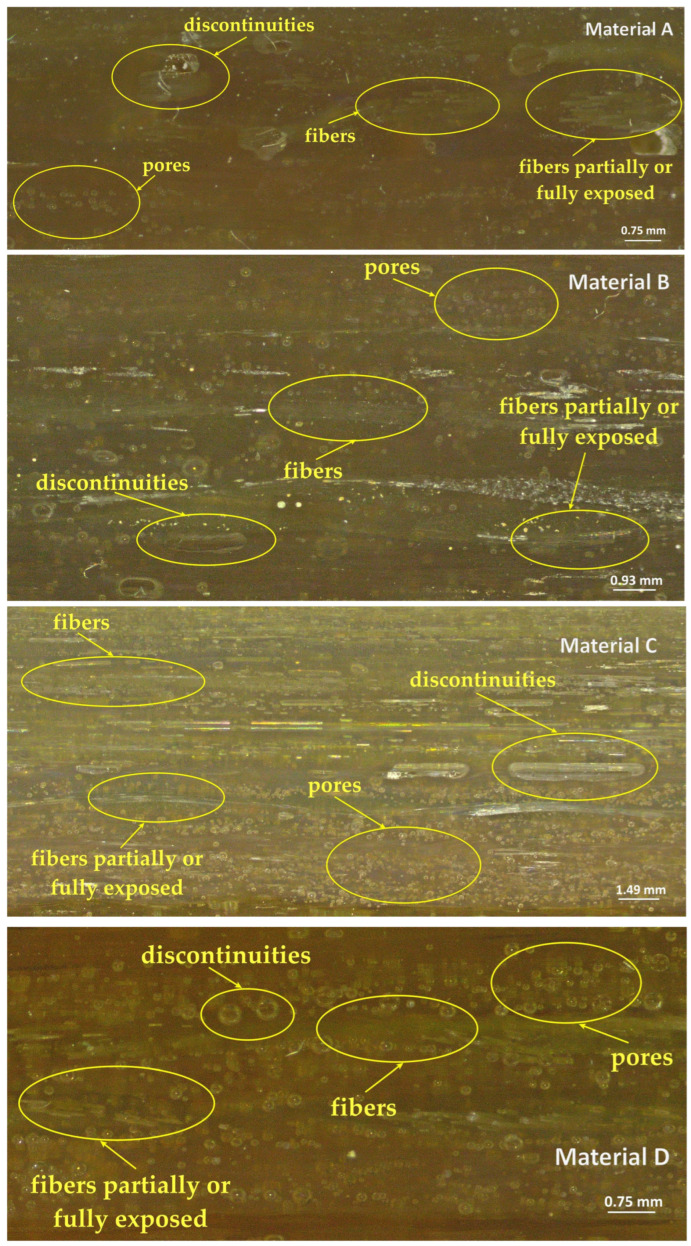
Specimen internal structure.

Microscopy specimens were prepared from each material type. The samples were polished on felt cloth using a 0.05 µm alumina suspension. Figure 8 presents the microscopic images of the four analyzed material types.

The obtained micrographs were further analyzed, and the proportions of each material in the specimens are presented in Table 6.

In all micrographs, the glass fibers were predominantly aligned longitudinally, indicating a manufacturing process in which the fibers were directionally oriented to enhance mechanical strength along that axis. The fibers appeared largely continuous and parallel, though certain areas exhibited disrupted or misaligned regions. In nearly all images, numerous small bubbles were visible, dispersed throughout the polymer matrix—some round, others elongated. These were manufacturing defects, most likely resulting from entrapped air during the resin casting or impregnation process.

Materials A and B exhibited regions of delamination or microcracking around the fibers, which may indicate poor adhesion between the glass fibers and the polymer matrix. Materials C and D showed the smallest air bubble sizes (5–30 μm), while Material A displayed medium-sized voids (10–50 μm) and Material B presented the largest bubble dimensions (10–100 μm). Material C demonstrated the most uniform fiber distribution, with well-aligned and densely packed fibers. Material D showed similar characteristics, although some irregularities in fiber arrangement were present. Material A exhibited a clear longitudinal orientation and a relatively uniform distribution. In contrast, Material B showed weak and locally disordered fiber alignment.

For structural applications (e.g., vehicle body components, load-bearing elements), Material C is clearly the most suitable composite. Materials A and B require improvements in the manufacturing process, such as enhanced degassing control, better fiber orientation, and more uniform resin impregnation.

Composite structure can be optimized to achieve a balance among stiffness, flexibility, weight, and durability by strategically orienting different layer types (e.g., mat and woven fabric) to control the directionality of mechanical strength, for instance, maximizing resistance along the fiber axis.

## 4. Conclusions

The fracture behavior of composite materials based on epoxy resin and glass fibers is a complex process governed by the interaction between the matrix (resin) and the reinforcement (fibers). These materials are widely used in structural applications because of their excellent strength-to-weight ratio.

A comparative analysis of the four material types, based on the results obtained in this study, revealed the following:

- Material A exhibited very low resilience, confirming its brittle nature and weak mechanical performance. It should be avoided in dynamic applications.
- Material B demonstrated a balanced combination of strength, deformability, and shock absorption capacity, making it a versatile material with wide-ranging applicability.
- Material C presented an interesting paradox: although it behaved in a brittle manner under flexural loading, it achieved the highest Charpy resilience. This may have been due to its ability to absorb energy over a localized zone or to an internal structure that responds differently to impact versus slow-loading conditions.
- Material D showed high mechanical strength but poor impact energy absorption, making it suitable for static loading applications, albeit potentially risky in dynamic environments.

The combination of a specific number of crosswise layers with a certain number of parallel layers—representing the novel aspect of this study—has yielded mechanical properties comparable to, or even exceeding, those reported by other researchers.

Based on the analysis of the results obtained in this study regarding the internal structure of glass-fiber-reinforced polyester composite materials, the following conclusions can be drawn:

- The three-layer composite (material A) is recommended for components with moderate strength requirements and low weight. It represents an optimal solution for simpler or less demanding parts, such as lightweight casings, decorative elements, or insulating panels.
- The five-layer composite (material B) is suitable for applications requiring increased strength and stiffness while still maintaining good weight control, for example, components used in the automotive sector (body panels), furniture parts, small boats, and similar applications.
- The seven-layer (or higher) composite (material C) is recommended for mechanically demanding parts that must withstand high loads, impacts, or intensive wear. Examples include structural components in aerospace, marine parts exposed to shocks, and high-performance sports equipment (such as snowboard decks or bicycle frames).

In the case of using these materials for tank manufacturing, material C is particularly well-suited for the main body of the tank—especially when the tank is pressurized or has structural load-bearing requirements.

Provided that manufacturing conditions and reinforcement ratios are consistently maintained, and strict quality control is observed throughout the production process (e.g., pressure, temperature, homogenization), there should be no significant behavioral variations across different product batches.

## Figures and Tables

**Table 1:** Architectural configuration and symbolization of the tested composite materials.

Material	Sample Group Symbol
Lower cylinder CRL 2 mat + 2# + 1||	A
Tank bottom 3T + 4M	B
Cylinder 2M + 1T; 2|| + 2# + 1||	C
Tank dome CRL	D

CRL—chemical-resistant lining, #—crosswise configuration layers, ||—parallel configuration layers.

**Table 2:** Comparative summary of the tensile behavior of the four studied materials.

Material	Strength (MPa)	Stiffness	Total Deformation	Behavior Type
A	Low (37)	Low	High	Ductile
B	Very high (114)	High	High	Elastic–rigid
C	Medium (72)	Medium	Medium	Slightly plastic
D	High (91)	Very high	Low	Brittle

**Table 3:** Comparative summary of the flexural behavior for the four studied materials.

Material	Maximum Load (N)	Extension	Failure Mode	Behavior Type
A	Low (200)	High	Ductile	Flexible, capable of withstanding large deformations
B	Medium (1990)	Medium	Semiductile	Moderate elastic behavior
C	Very high (12,650)	Low	Brittle	Rigid, unreliable under overload
D	High (720)	High	Pseudobrittle	Strong, yet susceptible to failure

**Table 4:** Impact toughness (KC) and fracture characteristics of composite materials.

Material	KC (J/cm^2^)	Behavior Type	Qualitative Impact Response
A	0.29	Very poor	Brittle under impact loading
B	1.25	Moderate	Reasonable impact performance
C	2.7	Very good	Excellent energy absorption capacity
D	0.68	Low	Lower impact toughness—likely a rigid structure with brittle response under shock loading

**Table 5:** Performance overview and application insights for the tested materials.

Material	Tensile Strength	Flexural Strength	Resilience (Impact Toughness)	Conclusions
A	Poor	Very poor	Very low	Does not offer reliability under either static or impact loading.
B	Excellent	Good	Moderate	Suitable for tanks exposed to variable loads.
C	Moderate	Very good (but brittle)	Excellent	Risky—may fail suddenly despite its toughness. Requires strict manufacturing control.
D	Very good	Very good	Low	Good for static tanks, but risky in environments where shocks or temperature fluctuations may occur.

**Table 6:** Proportion analysis on the specimens’ internal structure.

Material	CRL (Volume %)	MAT (Volume %)	Total (Volume %)	
			Resin	Glass Fiber
A	26.70	73.30	48.01	51.99
B	21.70	78.30	46.51	53.49
C	9.70	90.30	42.91	57.09
D	13.60	86.40	44.08	55.92

## Data Availability

The original contributions presented in this study are included in the article. Further inquiries can be directed to the corresponding author.
